# The role of iron in driving sex-biased differences in cancer

**DOI:** 10.1007/s10555-026-10340-3

**Published:** 2026-05-12

**Authors:** Aurosman Pappus Sahu, James R. Connor

**Affiliations:** Department of Neurosurgery, The Pennsylvania State University College of Medicine, Hershey, PA 17033 USA

**Keywords:** Iron, Sex differences, Tumor microenvironment, Tumor-associated macrophages, Ferroptosis

## Abstract

Sex is a critical determining factor in cancer incidence, pathogenesis, and outcome. These effects result from sexual differentiation influencing mechanisms that regulate both normal and cancer biology. Iron biology is sexually dimorphic in nature and plays a key role in tumorigenesis. Therefore, iron metabolism may be a key driver of sex-biased differences in cancer. In this article, we review and synthesize the existing literature examining iron’s role in driving sex differences in tumor incidence, progression, response to therapy, and overall survival in different cancer types. We also explore models that could be useful in studying iron metabolism–driven sex differences in tumorigenesis.

## Introduction

The role of transition metals in tumor progression has been an understudied area. This review focuses on iron, which is central to several physiological and cellular processes [1]. Along with its crucial role in oxygen transport, iron plays a direct role in gene regulation, metabolism, bioenergetics, and hormone synthesis. Cancer cells display a more aggressive utilization of iron, resulting in higher iron uptake than normal cells to support robust growth [2]. After the discovery of “Ferroptosis,” an iron-dependent cell death, however, the role of iron in tumor development is not as straightforward as initially thought. Cancer cells do need high iron to continue proliferating but need to regulate the amount of cellular iron to protect themselves from oxidative stress and ferroptosis [3, 4].

Iron metabolism is also sexually dimorphic. Females generally have lower systemic iron levels than males, which makes them more susceptible to iron deficiency anemia. In some tissues, males have an almost two to threefold higher amount of stored iron. Regarding blood iron, females have lower serum iron, serum ferritin, and hemoglobin than males [5]. These sex-biased differences are historically linked to blood loss through menstruation and conditions such as pregnancy. However, it has gradually become evident that these sex differences result from various additional factors such as genetic (presence/absence of Y chromosome), epigenetic, and hormonal differences. Sex chromosomes have an indirect effect on iron metabolism, as few key iron-regulatory genes are located on them. Instead, sex chromosomes mainly influence iron metabolism primarily through their role in determining gonadal development, which in turn regulates the production of sex hormones such as estrogen and testosterone. These hormones directly affect iron homeostasis, largely by modulating pathways involving hepcidin, and may also exert indirect effects through epigenetic regulation of iron-related genes. For instance, there is an inverse correlation between estrogen and serum ferritin; as a result, females accumulate more iron after menopause [6]. Yet, this increased iron in postmenopausal females is still considerably lower than the iron storage in age-matched males, indicating that the sex difference observed is not dependent on only estrogen [7]. Due to these explicit sex biases, males have been associated with increased cancer risks more than females, owing to their higher serum and tissue iron content. In this review, we examine the current literature describing how iron metabolism contributes to sex-biased differences across multiple cancer types, including glioblastoma, hepatic malignancies and colorectal cancer. We further explore the molecular and cellular mechanisms underlying these disparities, highlighting pathways that may be conserved across tumor types and that collectively drive sex-specific differences in cancer incidence, progression, and response to therapy.

The current standard of cutting-edge cancer treatments targets specific genetic or metabolic events that are involved in tumorigenesis. However, this approach fundamentally assumes that gene or protein target has the same therapeutic value regardless of all the other confounders that contribute to the heterogeneity among patients. With the emergence of several drugs targeting iron metabolism and ferroptosis in clinical trials to treat different types of cancers, there is a need to appreciate and take into consideration the sex-biased nature of iron metabolism both in the tumor microenvironment and on a systemic level.

### Systemic iron homeostasis

**Fig. 1:**
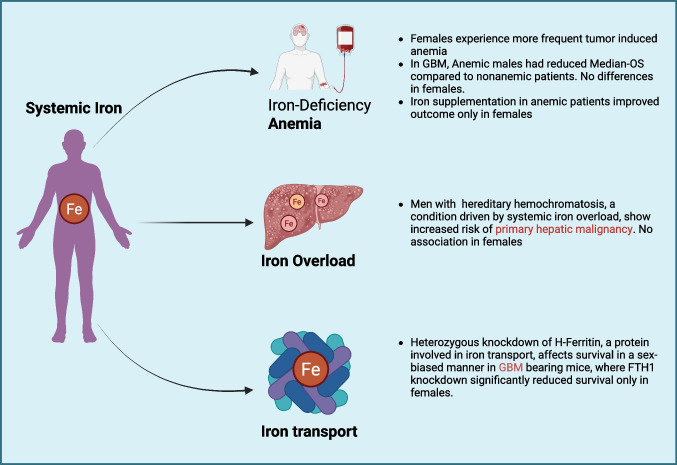
Overview of how systemic iron levels drive sex-biased differences in cancer incidence and survival. Anemia (indicating systemic iron deficiency), Iron overload, and deficiency of H-Ferritin (a protein involved in iron transport) all influence a significant facet of tumor progression (incidence or survival) in a sex-biased manner

Numerous epidemiological studies have shown a strong association between systemic iron levels and cancer [8–10]. These studies use well-established serum iron biomarkers such as circulating iron-binding proteins (TFRC and FTH1), transferrin saturation (TSAT), hemoglobin, hematocrit, total iron-binding capacity, and total serum iron levels to assess systemic iron levels and determine the correlation with cancer outcomes. Several diseases can cause an imbalance in systemic iron levels, leading to iron deficiency or overload. These iron imbalances can, in turn, affect tumor progression (Fig. 1).

Three key cell types are involved in regulating systemic iron levels. These are duodenal enterocytes, which absorb iron from the diet; macrophages, which recycle iron from red blood cells and other senescent cells; and hepatocytes, which act as a systemic iron reservoir and release iron to the blood when needed. The majority of iron transport is accomplished through the blood. The hepatocyte-synthesized hormone, hepcidin, plays a key role in downregulating iron release from tissues to the plasma [11]. Tumor-related inflammation can increase the production of hepcidin in the liver. The release of hepcidin can cause systemic iron deficiency by inhibiting ferroportin-mediated iron release from the macrophages and intestinal cells as well as iron deficiency in the TME by reducing iron release from the TAMs. Hence, clinically, functional iron deficiency-caused anemia is observed in cancer patients, which is brought about by the inflammatory nature of tumors and the side effects of cancer therapy [12]. Hepcidin is directly regulated by estrogen, a sex hormone, leading to explicit sex biases in hepcidin-mediated iron metabolism as well as inflammatory response. The serum hepcidin level is higher in males than females, which could lead to differences in tumor-induced functional iron deficiency [13]. There have been reports of sex differences in the onset of symptoms of cancer-related anemia, where males experience the symptoms of cancer-related anemia at a higher hemoglobin level, but females experience tumor-induced anemia more frequently than males [14].

Dietary iron exists in two primary forms: heme iron, derived from animal-based sources such as meat, poultry, and seafood, and non-heme iron, which is found in plant-based and fortified foods. Heme iron is more efficiently absorbed by the body and exhibits greater bioavailability compared to non-heme iron, making it a particularly influential component of dietary intake. Studies in colorectal cancer have shown that there are sex-biased associations of colorectal cancer risk with heme and non-heme iron uptake. The study, which included 450,105 participants, concluded that dietary non-heme iron reduced colorectal cancer risk in men, whereas heme iron showed a positive correlation with cancer risk. In females, however, dietary iron, irrespective of the food sources, did not show any association with colorectal cancer risk [15]. This study establishes the sex-biased connection between heme and non-heme dietary iron uptake and colorectal cancer risk. Further studies across a broader range of cancer types would be of interest to determine whether these patterns are consistent and to better understand the underlying mechanisms linking different forms of dietary iron to cancer susceptibility in males and females.

Systemic iron transport occurs through the plasma in a protein-bound form. Two of the most important proteins involved in iron transport are transferrin and ferritin. The primary function of ferritin is iron storage. However, the H subunit of ferritin (FTH1) can act as an iron delivery protein, transporting iron from one cell/tissue to another. As cancer cells need higher iron uptake to maintain proliferation, systemic FTH1 levels of the host can affect tumorigenesis and overall survival by impacting iron delivery to the tumor. Systemic ferritin levels are sex-biased, with males having higher cellular and serum ferritin than females [16]. Hence, systemic ferritin can contribute to sex biases in tumor progression and outcome. A recent study in a murine glioblastoma model showed that heterozygous knockdown of H-ferritin (FTH1) affects survival in a sex-biased manner, where FTH1 knockdown significantly reduced survival only in female mice [17].

There are several genetic and dietary predispositions that could lead to iron-related diseases. Most of these diseases include iron overload disorders and iron deficiency anemia. The current understanding of iron overload in tumorigenesis has been limited to its impact on oxidative stress-driven DNA damage and lipid peroxidation. It is widely established that tumors take up more iron by increasing the expression of TFRC and DMT1, resulting in an iron-rich cellular state, which is ideal for DNA damage, cellular ROS, and proliferation, all driving factors in tumorigenesis. However, how diseases like hemochromatosis, which cause systemic iron overload, affect tumor progression is yet to be fully elucidated. There is an increased risk for hepatocellular carcinoma in hemochromatosis patients, which is mainly driven by iron overload-induced damage to hepatic cells [18]. A clinical study showed that only men with hereditary hemochromatosis (HH) pathogenic variant (HFE p.C282Y) had a significantly increased risk of primary hepatic malignancy incidence and prognosis compared to wild type. However, no such association was seen in females [19]. This indicates that there is a sex-biased effect of systemic iron dysregulation on tumor progression.

There is a sex-biased association between anemia and tumor survival, recently demonstrated in glioblastoma. The study, which included 1346 glioblastoma patients, concluded that anemia was associated with reduced median overall survival (median-OS) compared to matched nonanemic patients only in males. Median hemoglobin levels were quantified for 6 months following diagnosis and used to classify patients as anemic or non-anemic according to the WHO guidelines (male 13 g/dL, female 12 g/dL). Female patients showed no association of anemia with median-OS. Iron supplementation is the standard treatment for anemic patients. However, in the context of cancer, longstanding arguments oppose supplementing iron in cancer patients, as it is commonly believed that iron may promote tumor growth and development. Iron supplementation in anemic GBM patients also had a sex-biased effect. Iron supplementation in anemic female GBM patients showed a positive trend toward increased median-OS, whereas no such association was observed in anemic males [20]. This study supports the need to evaluate the impact of iron supplementation on anemic patients following a diagnosis of GBM, assessing its influence on long-term prognosis in conjunction with standard-of-care treatments.

**Fig. 2:**
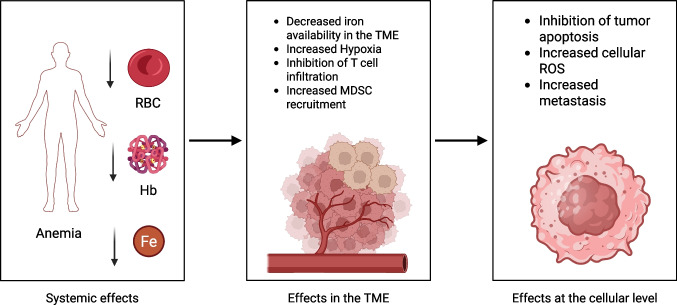
Overview of how anemia affects tumor progression: a classic hallmark of anemia is decreased red blood cells (RBC) and hemoglobin (Hb) in the blood. As a result, anemic patients have less oxygen delivered into the tumor. This initiates hypoxia-induced signaling, which remodels the TME to make it more immunosuppressive. On a cellular level, hypoxia induces increased oxidation stress in cancer cells and suppresses the pathways involved in cell death, as well as activating the signaling pathways that contribute to metastasis

The biological effect of anemia is related to oxygen delivery to the tumors and surrounding tissues. Hence, anemia could reduce tumor oxygenation, leading to tumor hypoxia and changes in tumor-associated phenotypes driven by the hypoxia-inducible factors (Fig. 2). Tumor-induced hypoxia could contribute to increased angiogenesis, reduction of host T cell anti-tumor immunity, reprogramming of tumor-associated myeloid cells, and treatment resistance [21]. Hence, it is not a surprising finding that anemia is unfavorably associated with different malignancies such as colorectal, breast, lung, and urologic cancers [20]. The data also raise the clinical questions of whether anemic patients should be supplemented with iron and if the decision should be based on patient sex. In GBM, studies have shown that only anemic female patients exhibit increased overall survival after iron supplementation, but further clinical trials need to be done in other cancer types for a universal association.

Sex-hormones play a significant role in systemic and tissue iron homeostasis, primarily by regulating the expression of hepcidin. Hepcidin contains a functional estrogen response element (ERE) half-site in its promoter region, enabling the estrogen receptor alpha to directly downregulate hepcidin mRNA transcription [22, 23]. The iron exporter FPN also contains an ERE and is directly regulated by estrogen, where E2 treatment leads to downregulation of FPN mRNA [24]. Similarly, estrogen can also regulate inflammation by inhibiting the NfkB pathway, hence indirectly reducing hepcidin levels [25]. Testosterone has also been shown to inhibit hepcidin production, thereby increasing iron release from the liver into the blood and stimulating erythropoiesis [26]. In conclusion, sex hormones could regulate tumor microenvironment iron homeostasis through direct transcriptional control of hepcidin and FPN, as well as indirect modulation via inflammatory signaling pathways, suggesting a mechanistic basis for sex-specific differences in tumor iron availability.

The existing literature shows strong indications that both iron deficiency anemia and systemic iron overload could affect tumor incidence and prognosis in a sex-biased manner. However, the mechanistic details of the intertwined role of genetic and hormonal factors and iron metabolism in tumor progression remain unexplored. Understanding the correlation between systemic iron levels and different types of cancer outcomes stratified by sex, as well as the underlying mechanism, can bring about significant breakthroughs in the field of cancer treatment and management. Although correlative evidence suggests that systemic iron markers such as hemoglobin, hematocrit, TSAT, and serum FTH1 influence cancer outcomes, establishing causality remains challenging. To interrogate the independent effects of systemic iron on cancer prognosis, epidemiological studies must rigorously account for confounders, such as chronic inflammation, liver diseases, metabolic syndromes, pre- and postoperative blood loss, and chemotherapeutic drugs, that could alter iron levels independent of the tumor.

### Cellular iron homeostasis

Systemic, tissue, and cellular iron metabolism acts in an intertwined manner. On a systemic level, the liver mainly controls iron homeostasis, whereas on a tissue level, macrophages control iron homeostasis. In the tumor microenvironment, tumor-associated macrophages (TAMs) locally recycle and store iron by phagocytosing senescent erythrocytes and necrotic tumor cells. The stored iron can then be released to the proliferating tumor cells either by exosomes, protein-bound form, or ferroportin-mediated free iron form, which can be later taken up by transferrin. There are clear sex differences in macrophage-iron handling in the TME. Mechanistically, estrogen negatively regulates hepcidin production, leading to increased ferroportin-mediated iron release in females [27]. Meanwhile, males have higher macrophage iron retention and storage. This results in a sex-biased difference in iron availability in the TME, bringing about functional changes in the different cell types present in the TME, such as neutrophils, dendritic cells, and cancer-associated fibroblasts (CAFs). Macrophage polarization significantly dictates iron storage and release dynamics in the TME. While proinflammatory M1 macrophages typically sequester iron from the environment, anti-inflammatory M2 macrophages are characterized by enhanced iron release [28]. Similarly, high macrophage iron levels induce M1 polarization whereas lower iron induces M2 polarization. Although TAMs are traditionally viewed as having an M2-like phenotype, this generalization overlooks their inherent transcriptional heterogeneity. In reality, the TAM population is a complex spectrum; while predominantly M2-heavy, it retains a significant proportion of M1-like transcriptional states. Sex hormone–mediated signaling can influence macrophage polarization; however, whether they promote any specific polarization state is highly context dependent. For example, estrogen can inhibit M2 polarization in HCC via the ER-beta pathway, whereas it can promote M2 macrophages in bladder cancer via the ER-alpha pathway [29, 30]. Androgen signaling on the other hand mostly favors M2 type polarization and contributes to a pro-tumor TME [31]. In the TME, the cellular state of TAMs is the result of the combination between iron-mediated metabolic reprogramming and hormone/cytokine-mediated transcriptional reprogramming. To completely understand how these two mechanisms interact with one another, proper model systems need to be designed where only one of the two mechanisms can be manipulated while keeping the other unchanged. Lower iron availability is interlinked with hypoxic signaling in the TME. Typically, hypoxia is a result of tumor growth outpacing angiogenesis, resulting in reduced blood flow and a lack of oxygen and iron. Under hypoxia, tumor cells upregulate DMT1 and TFRC to get more iron in response to cellular iron deficiency. Hypoxia-mediated metabolic reprogramming of the TME makes it more immunosuppressive by impairing the functions of cytotoxic T cells, NK cells, and dendritic cells. Hypoxia-mediated signals also activate fibroblasts into cancer-associated fibroblasts, which are involved in several pro-tumor processes such as ECM remodeling and promotion of angiogenesis [32].

Differences in iron status in a single cell population can cause significant heterogeneity in the TME. Recently, researchers discovered an iron-rich CAF called “FerroCAFs,” which accumulate high iron by hemoglobin degradation. These ferroCAFs have increased myeloid cell-associated cytokines expression, creating an immunosuppressive microenvironment by recruiting myeloid-derived suppressor cells (MDSCs) [33]. Similarly, an iron-rich subpopulation of TAMs (iTAMs) has been discovered that promote angiogenesis and immunosuppression in the tumor microenvironment [34]. The spatial distribution of these iron-rich CAFs and TAMs can remodel their immediate periphery in the TME. At this point, it is unknown whether the distribution and frequency of these iron-rich cells in the TME depend on sex. However, considering the sex-biased nature of macrophage-mediated iron distribution at a tissue level, we can hypothesize that there might be a potential link between the two. Given that both TAM and CAF activation and recruitment is directly regulated by sex hormones such as estrogen, there is a need for hypothesis-based investigation to uncover how the sex-biased interplay between hormonal signaling and iron availability contribute to heterogeneity in the tumor microenvironment.

At the cellular level, iron can be taken up in protein-bound form (Fe3 +) by receptor-mediated endocytosis or in free iron form (Fe2 +) by divalent metal transporters (DMT-1). However, the transferrin receptor mediates most cellular iron uptake, which binds to holo-transferrin and facilitates iron uptake by a clathrin-mediated endocytic pathway. Homeostatic iron-regulator protein (HFE) regulates iron uptake by binding to transferrin receptors (TFRs), decreasing TFR affinity to transferrin and reducing iron uptake. After being taken up into the cell, the iron further contributes to the labile iron pool or is stored within ferritin. The proteins involved in cellular iron homeostasis are normally dysregulated in cancer cells and can affect tumor phenotypes in a sex-biased manner. A recent bioinformatics study from TCGA by Nesterova et al. has shown that HFE has a sexually dimorphic role in survival in glioblastoma patients. This study, which included microarray gene expression and clinical data from all IDH-wild type primary GBM patients, interrogated the correlation of tumor tissue HFE expression with overall survival, stratified by sex. High HFE expression correlated with reduced survival in female patients [35]. Further studies in a syngeneic *in vivo* GBM model by Troike et al. showed that cellular HFE levels could affect tumor proliferation and overall survival in a sex-biased manner by modulating tumor cell-intrinsic and extrinsic factors. Intracranial injection of HFE-suppressed cells caused an increase in overall survival in a sex-biased manner (more prominent in females) by inducing cellular apoptosis (tumor intrinsic) and attenuation of natural killer cells and CD8 T cells (tumor extrinsic) [36]. These studies indicate that tumor iron status (including iron uptake and retention) can affect overall tumor growth (survival) and the local TME in a sex-biased manner. Fluctuations in tumor iron levels can induce upregulation of cytokine secretion from the cancer cells, which can remodel the immediate niche of the tumor. Cancer stem cells are especially highly dependent on iron and play a pivotal role in remodeling the local TME to promote tumor growth [37]. The heterogeneity of the iron status of cancer cells in a solid tumor can be brought about by stemness, hypoxia, and location of the cancer cell in the tumor (core or periphery). Studying the interactions between cancer cell populations with different iron signatures and how these populations are affected by the patient’s sex can further the field significantly. With the advances in spatial technologies, it will be possible to study the immediate neighboring cells of an iron-rich TAM or CAF and understand how these cells form their unique niche and alter the TME dynamics. Especially with single-cell resolution spatial transcriptomics platforms, it is possible to compare the distribution and frequencies of such unique iron-rich/iron-poor (hypoxic) niches in male and female tumors and how they change the nearby tumor cells and other immune cells.

**Fig. 3:**
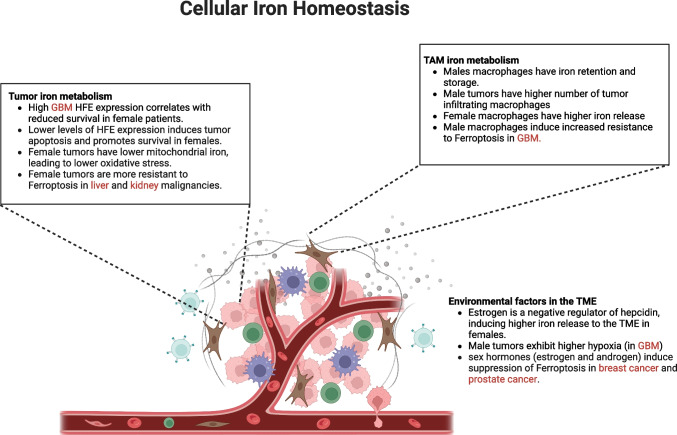
Overview of how cellular iron metabolism in the TME drives sex-biased differences in cancer. TAMs show sex-biased iron metabolism, with sex-biased differences in iron uptake, storage, and release. As a result, being the primary cells that handle iron distribution in the TME, TAMs regulate iron availability to cancer cells and other cells in a sex-biased manner. In cancer cells, sex differences in iron uptake and mitochondrial metabolism drive sex-biased phenotypes. Finally, the presence of sex hormones in the TME can affect the overall iron distribution in the TME by regulating genes involved in iron metabolism, such as hepcidin

Cellular and mitochondrial iron metabolism are pivotal pathways for tumorigenesis, and they show significant sex differences. Females have lower cellular and mitochondrial iron than males, leading to differences in oxidative stress and mitochondrial function. This difference in mitochondrial iron levels is due to lower transferrin receptor and mitoferrin expression, resulting in lower mitochondrial iron uptake and storage in female mitochondria. These effects are most prominent in liver tissue, given its importance for systemic iron homeostasis. Cumulatively, these differences in cellular and mitochondrial iron metabolism can affect ferroptosis in a sex-biased manner. A study done in liver cells showed that male hepatocytes are more vulnerable to ferroptotic inducers than females. This is because of elevated mitochondrial Fe^2+^ and mitochondrial ROS levels in male hepatocytes [38]. Another study in kidney cells showed sex differences in ferroptosis, where female renal cells confer significant resistance to ferroptosis compared to males via an NRF2-dependent mechanism [39]. Mechanistically, sex hormones can upregulate the expression of two ferroptotic suppressor proteins, MBOAT1/2. A combination of estrogen receptor or androgen receptor antagonists, along with ferroptosis inducers, has shown promising results in inhibiting the growth of ER + breast cancer and AR + prostate cancer [40]. The intrinsic sex differences in iron storage observed in tumor cells, along with the regulation of phospholipid-modifying enzymes such as MBOAT1/2 by sex hormones, indicate a strong sex bias in the regulation of ferroptosis in cancer biology. With the increasing emergence of different ferroptosis inducers as potential anti-tumor drugs, it is essential to decipher the role of the patient’s sex in tumor-associated ferroptosis. Overall, the existing literature provides strong evidence that iron can drive sex differences in tumorigenesis via different cell types in the TME and is heavily interlinked to the sex hormones (Fig. 3).

### Model systems to study sex differences in tumor iron metabolism

In the past decade, several clinical and preclinical studies have addressed how the sexually dimorphic nature of iron metabolism could affect tumor incidence, progression, overall survival, and response to treatment [41]. However, a few key differences in iron physiology between murine models and humans can limit the translatability of preclinical studies in mice or rats to clinical trials. The most prominent of these differences is the absence of blood loss through the menstrual cycle in rodents. Nonetheless, there are hypotheses that can be tested in animal models. For example, we can explore how changes in systemic iron affect tumor progression. To understand how diseases that alter systemic iron levels, such as anemia or hemochromatosis, affect tumorigenesis, the systemic iron condition can be mimicked in mice by dietary or genetic modifications. Iron deficiency anemia can be modeled in mice by feeding them an iron-deficient diet. Once the mice are anemic, any specific tumor can be induced in the anemic mice to understand how anemia affects tumor growth, survival, and response to therapy, as well as iron supplementation in anemic male and female mice. Similarly, mice can be kept on an iron-rich diet to model higher iron conditions. H67D mutation is the mouse equivalent of the human H63D mutation that impacts survival outcome. Hence, H67D-mutatated mice can be used as a model to study how tumors develop in the presence of this mutation [42]. However, significant differences between human and murine pathophysiology need to be considered, such as the much shorter lifespan of mouse erythrocytes than that of humans. Mice have more active erythropoiesis and can mount a much more intense reticulocyte response to inflammatory stimuli and cancers [43].

Another important question to address is the therapeutic role of iron supplementation and chelation in cancer treatment. It has become increasingly clear that iron plays a multifaceted role in tumor progression, and altering the iron availability to cancer cells can be used to make cancer cells vulnerable to different types of cell death. There have been promising developments in using iron chelators to deprive cancer cells of iron [44, 45]. Similarly, there is promise in using iron supplementation in conjugation with ferroptosis inducers to increase the efficacy of the ferroptotic drugs. The effectiveness of these drugs targeting iron metabolism in the TME can be validated using mice models representing both sexes. Owing to the sex-biased nature of tumor iron metabolism, it can be hypothesized that drugs targeting iron metabolism will have a sex effect in both effectiveness and potential side effects.

## Conclusion

Iron is an essential nutrient and plays an important role in cellular metabolism. It is also required to support the rapid growth of cancer cells during tumorigenesis, making it a pivotal player in cancer biology. Due to the sexually dimorphic nature of iron metabolism, it can play an important role in driving sex-biased differences in cancer incidence, progression, and outcome. While our current review focuses exclusively on iron, we acknowledge that some observed sex-biased effects may be influenced by interactions between iron metabolism and other transition metals. Although being a relatively new field, the existing literature still provides compelling evidence that systemic and cellular iron status can influence overall survival, immune infiltration, and response to therapy (such as ferroptosis) in a sex-biased manner. It is difficult to provide a data-based unifying concept for a sex-biased role of iron in cancer. Overall, on a systemic level, females generally exhibit lower baseline iron and stronger activation of NRF2-dependent antioxidant pathways, including GPX4 and FSP1, resulting in reduced sensitivity to ferroptosis. However, during cancer, these differences do not necessarily manifest as lower ROS or lipid peroxidation levels in female tumors, but rather as an enhanced capacity to buffer oxidative stress and suppress ferroptosis–induced cell death. Whereas in males, there is elevated oxidative stress, and comparatively weaker activation of NRF2-dependent antioxidant defenses. This results in a reduced buffering capacity against ferroptotic stress in tumors. We can conclude that the existing literature emphasizes the need to develop sex-biased study designs and treatment strategies that target iron-mediated vulnerabilities, such as ferroptosis, in cancer.

## Data Availability

No datasets were generated or analysed during the current study.
